# Ductal Carcinoma In Situ of the Breast in Sclerosing Adenosis Mimicking Microinvasive Ductal Carcinoma: A Rare Case Report

**DOI:** 10.1002/ccr3.9639

**Published:** 2024-12-12

**Authors:** Yu‐Kai Chang, Yuan‐Yuei Chen, Zhi‐Jie Hong, Guo‐Shiou Liao, Jyh‐Cherng Yu

**Affiliations:** ^1^ Department of Surgery Tri‐Service General Hospital, National Defense Medical Center Taipei Taiwan; ^2^ Department of Pathology Tri‐Service General Hospital, National Defense Medical Center Taipei Taiwan; ^3^ Division of Traumatology, Department of Surgery Tri‐Service General Hospital, National Defense Medical Center Taipei Taiwan; ^4^ Division of General Surgery, Department of Surgery Tri‐Service General Hospital, National Defense Medical Center Taipei Taiwan

**Keywords:** breast cancer, case report, ductal carcinoma in situ, histochemical stains, Sclerosing adenosis

## Abstract

Sclerosing adenosis (SA) is a subtype of adenosis characterized by proliferative adenosis and stromal sclerosis with distortion of the terminal ductal lobular unit. Although SA is the most prevalent benign breast condition among middle‐aged women, it may be associated with a two‐fold increase in breast cancer risk. Microscopic findings of ductal carcinoma in situ (DCIS) in a SA (SA‐DCIS) may mimic microinvasive carcinoma or even invasive carcinoma, which may result in overtreatment by a breast surgeon. Therefore, pathological evaluation must be performed carefully in such cases to establish a definitive diagnosis. Herein, we present a rare case of SA‐DCIS that mimicked microinvasive ductal carcinoma. A 51‐year‐old woman was diagnosed with high‐grade DCIS of the left breast, highly suspicious for microinvasion, based on core biopsy. She underwent mastectomy of the left breast and prophylactic mastectomy of the right breast for personal reasons after imaging evaluation, including mammography and ultrasonography. However, the final diagnosis was bilateral intermediate‐grade DCIS, characterized by the presence of intraductal carcinoma cells exhibiting enlarged hyperchromatic nuclei and a high nuclear‐to‐cytoplasmic ratio within the mammary ducts. However, the diagnosis was incompatible with the initial pathological results. A‐DCIS has pathological features similar to those of microinvasive or invasive carcinomas. Thoroughly evaluating biopsy findings of lesions to distinguish between SA‐DCIS and invasive carcinoma is crucial to prevent overdiagnosis and unnecessary management in patients with DCIS. Patients diagnosed with SA‐DCIS are at a higher risk of developing bilateral breast cancer than those without SA‐DCIS.

**Abbreviations**
CK14: cytokeratin 14
DCIS: ductal carcinoma in situ
ER: estrogen receptor
HER‐2: human epidermal growth factor receptor‐2
N/C ratio: nuclear‐cytoplasmic ratio
PR: progesterone receptor (PR)
SA: sclerosing adenosis
SA‐DCIS: ductal carcinoma in situ developed from sclerosing adenosis

**Summary**

- Sclerosing adenosis is associated with a higher risk of breast cancer.
- The histopathological features of ductal carcinoma in situ in sclerosing adenosis are similar to those of microinvasive or invasive ductal carcinoma.
- Pathological evaluation is crucial for diagnosing and differentiating sclerosing adenosis involving ductal carcinoma in situ from other conditions to prevent unnecessary overtreatment.

## 1 Introduction

Sclerosing adenosis (SA) is a benign breast condition, mostly prevalent in women aged 45–55 years [1]. It is a subtype of adenosis characterized by proliferative adenosis and stromal sclerosis with distortion of the terminal ductal lobular unit. SA can present as round, punctate, or pleomorphic microcalcifications on mammography. SA is diagnosed in 27.8% of all benign biopsies on histopathological examination [1]. It can mimic malignancy when presenting with suspicious features, such as an irregular mass or focal areas of shadowing without a distinct mass. However, there are no specific features for diagnosing SA. Microscopically, SA shows lobulocentric proliferation in both epithelial and myoepithelial cells, and the complex proliferative alterations make it difficult to differentiate it from invasive carcinoma. Furthermore, SA is associated with a two‐fold higher risk of breast cancer [1]. Ductal carcinoma in situ (DCIS), which develops from SA (SA‐DCIS), may mimic microinvasive or invasive carcinoma on microscopic examination, leading to overdiagnosis and overtreatment. Pathological evaluation using immunohistochemical staining is important for diagnosis. Herein, we present a rare case of SA‐DCIS that mimicked microinvasive ductal carcinoma.

## 2 Case Presentation

**FIGURE 1:**
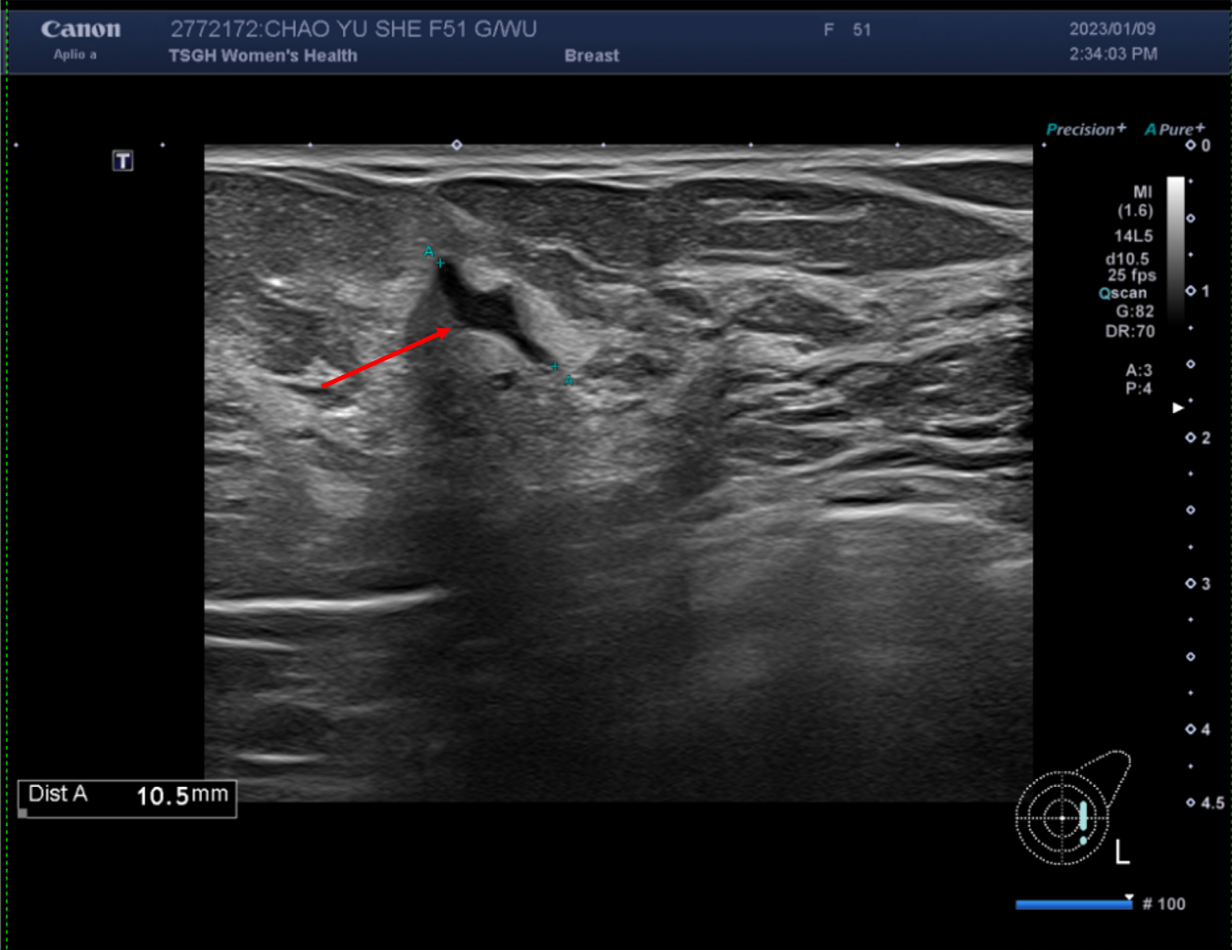
Sonography of the breast. A 10.5 mm lobular mass with poorly defined margins at the 3 o'clock position of the left breast (red arrow).

A 51‐year‐old woman complained of pain in the left breast for 1 week without a palpable mass. She visited a local hospital for regular breast follow‐ups over the past few years. She had a history of lung adenocarcinoma diagnosed following a wedge resection of the left upper lobe of the lung in May 2020 and again in 2023. She also had a diagnosis of leiomyoma after a robotic‐assisted laparoscopic subtotal hysterectomy in July 2014. She denied any history of smoking or drinking habits. There was a family history of lung cancer, specifically in her elder sister. Physical examination revealed multiple bilateral breast nodules. No skin dimpling, nipple discharge, or palpable nodes were observed in the axillary or neck regions. Laboratory studies revealed no specific abnormalities, and tumor markers, including CEA, CA‐153, and CA‐199, were within the normal range. Sonography of the left breast revealed a 10.5‐mm lobular mass with a poorly defined margin at the 3 o'clock position. The BI‐RADS category was 4A (Figure 1), indicating low suspicion of malignancy and requiring a biopsy.

## 3 Diagnostic Assessment and Therapeutic Intervention

**FIGURE 2:**
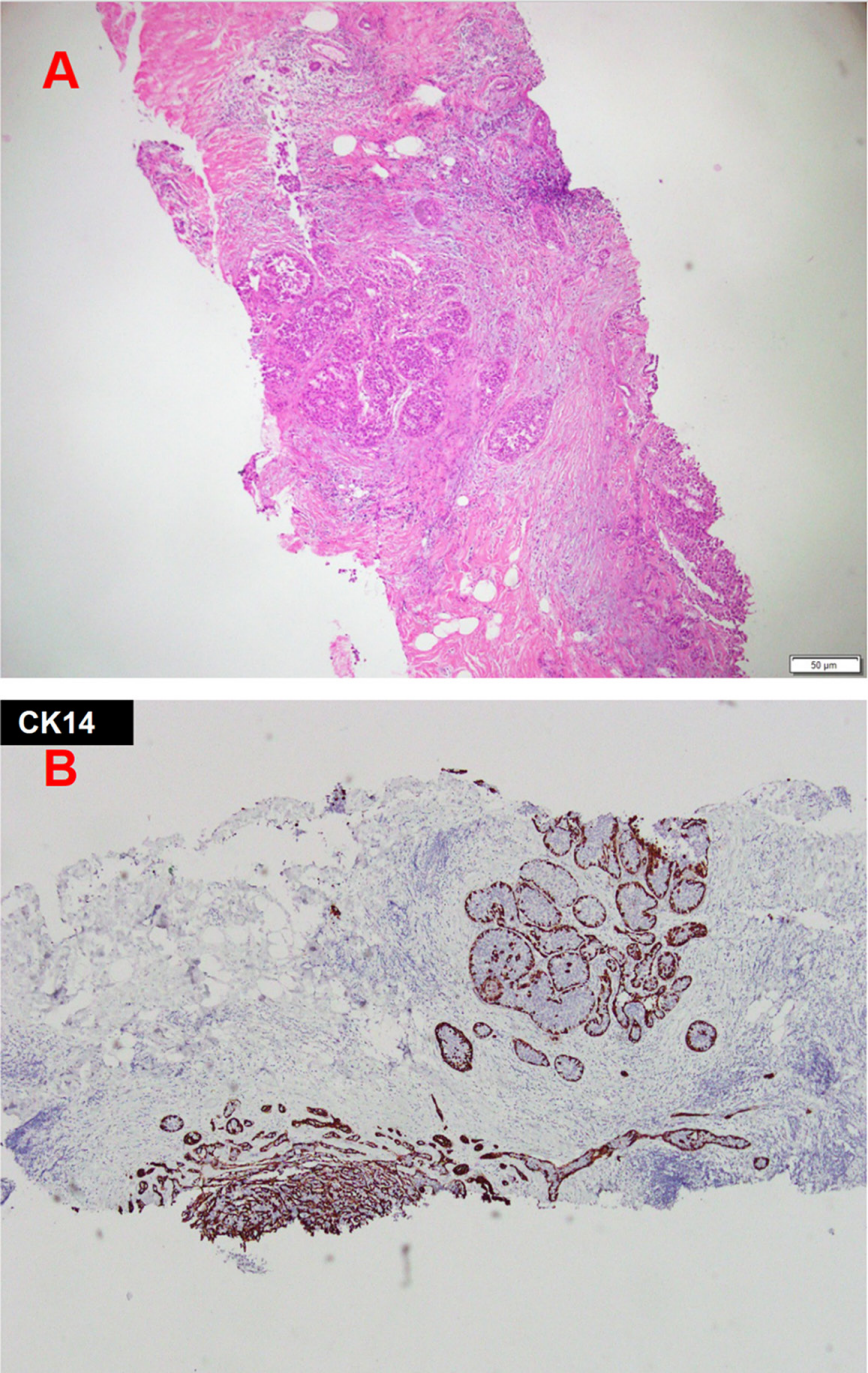
Sono‐guided core needle biopsy of the left breast. (A) High‐grade ductal carcinoma in situ of the breast tissue, suspected for microinvasion (H&E, 40×). (B) Pre‐surgery Cytokeratin 14 analysis (CK14 IHC, 40×).

**FIGURE 3:**
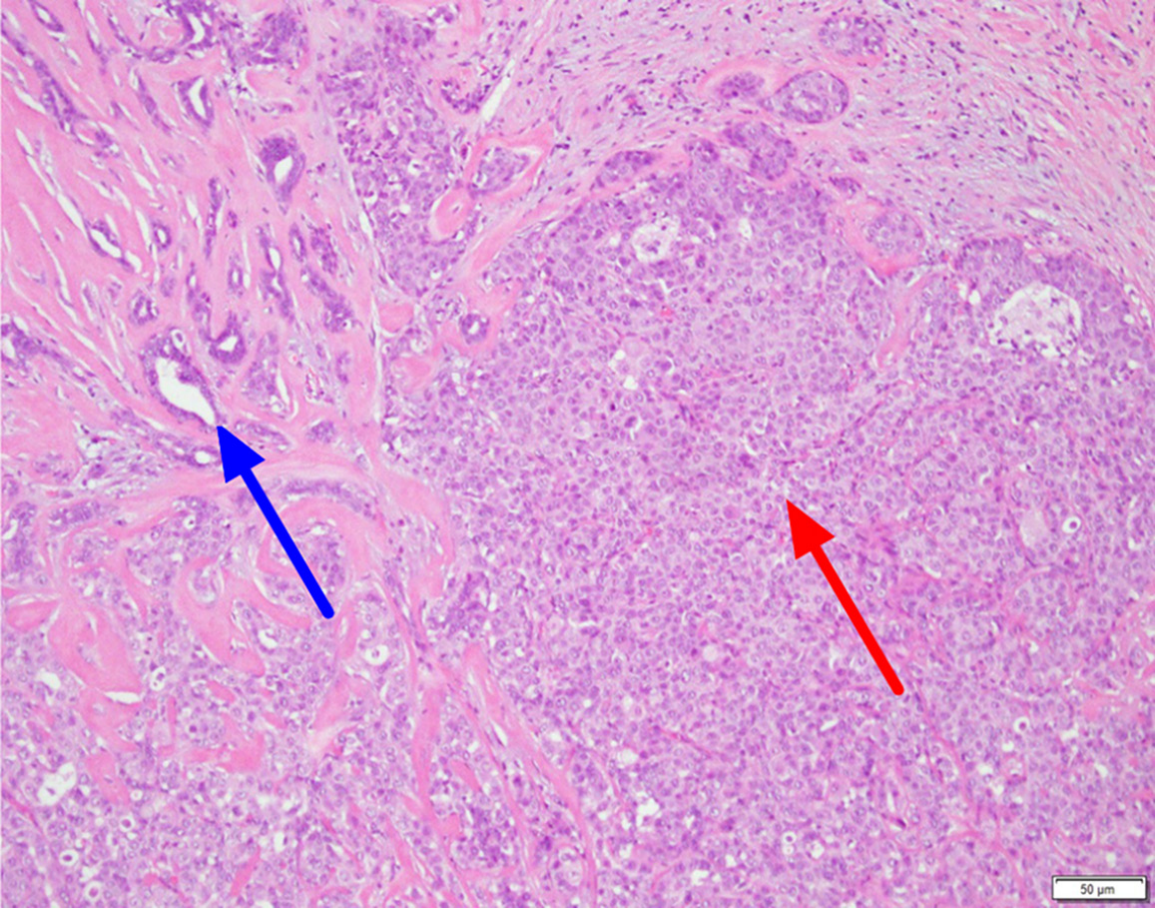
Mastectomy of the left breast (H&E, 100×). Ductal carcinoma in situ (red arrow) in the intermediate grade of breast tissue is characterized by intraductal carcinoma cells with enlarged hyperchromatic nuclei and a high nuclear‐cytoplasmic ratio within the mammary ducts. The cells around the carcinoma in situ show histopathological features, including sclerotic lobules that are significantly associated with invasive carcinoma (blue arrow).

**FIGURE 4:**
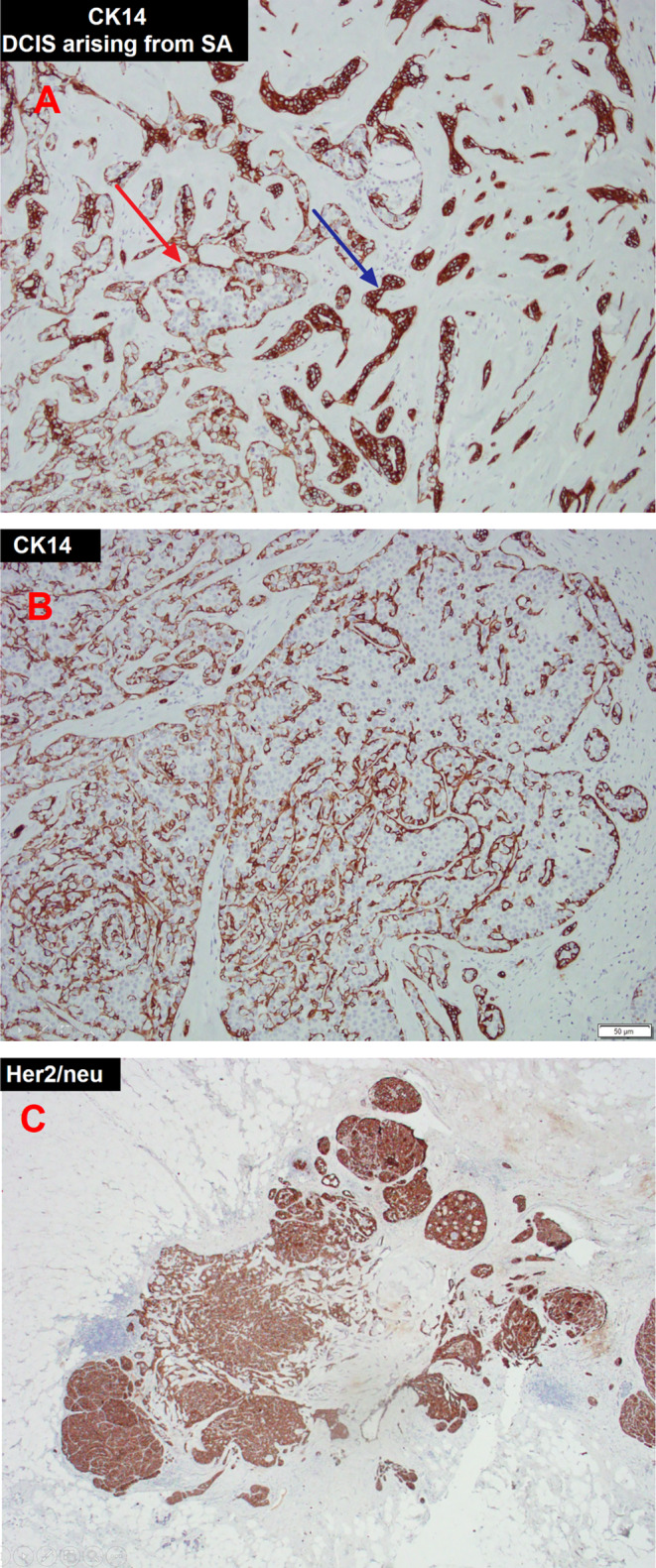
Mastectomy of the left breast. (A) Ductal carcinoma in situ (DCIS) arising from sclerosing adenosis (CK14 IHC, 100×). Immunohistochemical staining for CK14 performed on the section highlights the myoepithelial cells (brown chromogen) surrounding the carcinoma cells, indicating ductal carcinoma in situ (red arrow). Sclerosing adenosis consists of enlarged and distorted lobules containing duplicated and crowded acini with prominent myoepithelium and stromal fibrosis. Myoepithelial cells within the sclerosing adenosis are stained with CK14 (blue arrow). (B) DCIS (CK14 IHC, 100×). Proliferation of epithelial cells with a high N/C ratio in the breast ductal lobular unit confined to the myoepithelial basement membrane. (C) Her‐2 test (Her‐2 IHC, 40×).

Pathological examination of core needle biopsy under sonography revealed DCIS, which was highly suspicious for microinvasion (Figure 2). We planned to perform either a breast‐conserving surgery or a mastectomy of the left breast. However, the patient requested mastectomy of the left breast and prophylactic mastectomy of the right breast due to personal concerns; therefore, these procedures were performed. The resected specimen showed cells with enlarged hyperchromatic nuclei and a high nuclear‐to‐cytoplasmic ratio (N/C ratio) in the mammary ducts (Figure 3). Both breasts revealed DCIS. SA is characterized by enlarged acini that become slightly distorted by surrounding stromal fibrosis, with preserved two‐cell layers (inner epithelial and outer myoepithelial cells). Immunohistochemical staining revealed cytokeratin 14 (CK14) staining with preserved peripheral myoepithelial cells, indicating DCIS involving SA (Figure 4). Immunohistochemical staining showed estrogen receptor (ER) positivity, progesterone receptor (PR) positivity, strong human epidermal growth factor receptor 2 (HER‐2) positivity, and an increased proliferative index Ki‐67 (20%). The final diagnosis was bilateral DCIS, intermediate‐grade, pTisN0, and stage 0. At the time of writing this manuscript in September 2023, the patient was undergoing regular follow‐up in the outpatient department every 6 months. No evidence of recurrence or metastasis was observed during these follow‐ups.

## 4 Discussion

SA‐DCIS has histopathological features similar to those of microinvasive ductal carcinoma and invasive ductal carcinoma [2], which may lead to overdiagnosis or overtreatment. In this patient, sonography of the left breast revealed a lobular mass with a poorly defined margin at the 3 o'clock position with the BI‐RADS category 4A [3]. BI‐RADS category 4A suggests low suspicion of malignancy and requires a biopsy. A core needle biopsy of the left breast showed high‐grade DCIS, highly suggestive of microinvasion. Mastectomy and breast‐conserving therapy (BCS) are viable options for women with DCIS. Mastectomy has a high success rate, with > 98% of patients cured of DCIS. In contrast, BCS has certain advantages, such as lower postoperative complication rates. However, it carries a slightly higher risk of local recurrence than mastectomy [4]. DCIS with microinvasion (DCIS‐MI) is similar to invasive diseases and small‐volume IDC and has excellent prognoses with high rates of overall survival [5]. Patients with DCIS‐MI should receive nodal staging and adjuvant chemotherapy. Radiotherapy should be administered to patients with node‐positive diseases [6]. In the present case, the patient underwent mastectomy and contralateral prophylactic mastectomy. Histopathological examination of the resected specimen revealed DCIS arising from SA (Figure 4) instead of an invasive or microinvasive ductal carcinoma, which was not compatible with the core biopsy results. Involvement of SA by DCIS may be misdiagnosed as invasive carcinoma because of extensive DCIS with colonization of sclerotic lobules and accompanying periglandular lymphocytes, mimicking high‐grade invasive carcinoma [7]. A previous case report suggested that DCIS developing in SA has characteristics similar to those of invasive ductal carcinoma [8]. Moreover, DCIS, regardless of the involvement of SA, may present occult invasive carcinoma [9]. Careful histopathological examination of multiple sections of the right breast revealed DCIS. The characteristics of cancerization in SA (e.g., SA‐DCIS) and invasive carcinomas are similar [10]. Immunohistochemical staining for CK14 highlighted the presence of myoepithelial cells in carcinoma in situ. In our case, the mastectomy specimen revealed the proliferation of epithelial cells with a high N/C ratio within the mammary ducts, comprising myoepithelial and luminal epithelial cells.

A retrospective study indicated that patients with SA‐DCIS have a higher risk of developing bilateral breast cancer than those without SA‐DCIS (38% vs. 13%, *p* < 0.01). Therefore, careful postoperative imaging evaluation of the contralateral breast lesions is recommended for patients with SA‐DCIS [11]. Systemic adjuvant treatment, except for hormone therapy after mastectomy, is not required in patients with DCIS [12]. The histopathological reports between core biopsy and mastectomy were incompatible in this case. Pathologists must avoid overdiagnosis by differentiating SA‐DCIS and SA‐IDC using immunohistochemical staining, such as CK14, to identify focal myoepithelial cells.

## 5 Conclusion

The pathological features of SA‐DCIS are similar to those of invasive carcinomas. Immunohistochemical staining for CK14 plays an important role in distinguishing myoepithelial cells from SA. Therefore, it is important for pathologists and clinicians to carefully evaluate biopsy findings of the lesion to differentiate between DCIS involving SA and invasive carcinoma to prevent overdiagnosis, which may lead to overtreatment of patients with in situ disease. Patients diagnosed with SA‐DCIS by core biopsy of a unilateral breast are at a higher risk of developing bilateral breast cancer than those without SA‐DCIS. Therefore, these patients require careful evaluation and follow‐up to determine their risk of developing contralateral breast cancer.

## Author Contributions

**Yu‐Kai Chang:** data curation, writing – original draft. **Zhi‐Jie Hong:** resources, supervision. **Yuan‐Yuei Chen:** data curation, resources. **Guo‐Shiou Liao:** resources, supervision. **Jyh‐Cherng Yu:** resources, supervision, writing – review and editing.

## Ethics Statement

The study protocol was approved by the Ethics Committee of Tri Service General Hospital (Approval No. B202315121).

## Consent

Written informed consent was obtained from the patient for publication of this report and any accompanying images.

## Conflicts of Interest

The authors declare no conflicts of interest.

## Data Availability

The authors confirm that the data supporting the findings of this study are available within the article.
